# Radiolabeled Trastuzumab Solid Lipid Nanoparticles for Breast Cancer Cell: in Vitro and in Vivo Studies

**DOI:** 10.1021/acsomega.2c03023

**Published:** 2022-08-19

**Authors:** Emre Ozgenc, Merve Karpuz, Ege Arzuk, Marta Gonzalez-Alvarez, Marival Bermejo Sanz, Evren Gundogdu, Isabel Gonzalez-Alvarez

**Affiliations:** † Department of Radiopharmacy, Ege University, 35040, Izmir, Turkey; ‡ Department of Radiopharmacy, Izmir Katip Celebi University, 35620, Izmir, Turkey; § Department of Toxicology, Faculty of Pharmacy, Ege University, 35040, Izmir, Turkey; ∥ Department of Pharmacokinetics and Pharmaceutical Technology, Miguel Hernandez University, San Juan de Alicante, 03550 Elche, Alicante, Spain

## Abstract

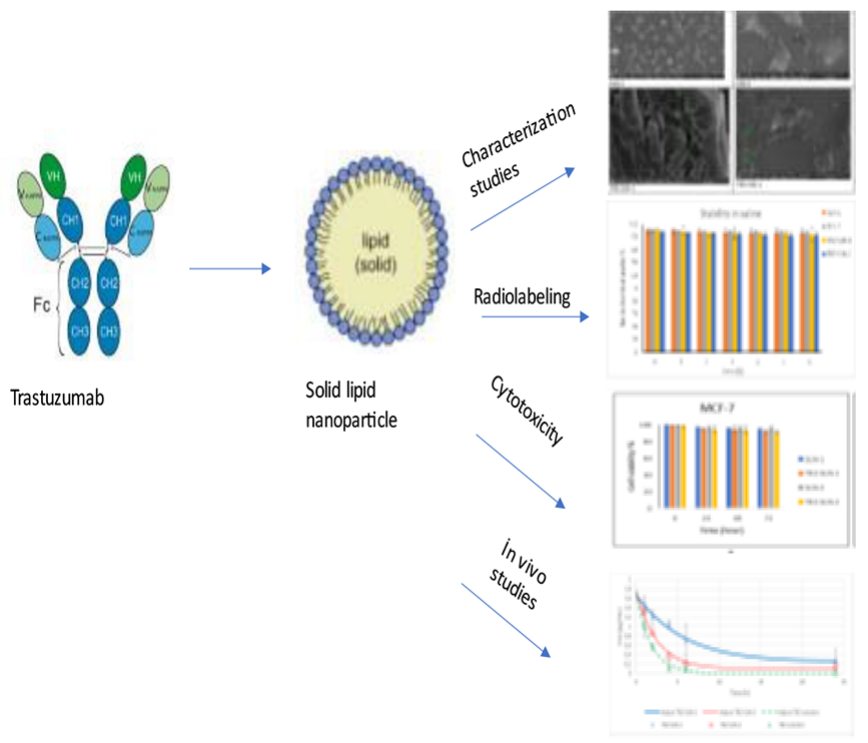

Radiolabeled trastuzumab (TRZ) loaded solid lipid nanoparticles (SLNs) were prepared by high shear homogenization and sonication techniques. The apoptosis mechanism of TRZ-SLNs was studied only with the MCF-7 cell line, while the cytotoxicity and cell binding capacity were investigated using breast cancer cells (MCF-7 and MDA-MB-231) and the human keratinocyte cell line (HaCaT). The particle sizes of TRZ-SLNs were found to be below 100 nm, and they possessed a negative charge. The high radiolabeling efficiency and good radiolabeling stability in saline and a cell culture medium were obtained in the results of radiolabeling studies. According to the in vitro studies, TRZ-SLNs were found to be biocompatible, and they effectively induced apoptosis in MCF-7 cells. After the parenteral injection of TRZ-SLNs into rats, a sustained release profile in blood circulation was achieved compared with free drug solution by the evaluation of pharmacokinetic parameters. As a conclusion, the study reveals that Technetium-99m (^99m^Tc radiolabeled) TRZ loaded SLN formulations could be promising theranostic agents based on their characterization profiles, in vitro cellular uptake and apoptosis induction capacity, and in vivo pharmacokinetic profiles.

## 1 Introduction

Breast cancer, the most common cancer type, is one of the most fatal diseases after heart disease in women. About 627 000 female deaths from breast cancer were reported in 2018, accounting for about 15% of total deaths among women.^1^ However, this condition is expected to overcome heart disease and become the most common cause of death in the coming years.^2^ Breast cancer has various clinical features, and it may be sensitive to treatment. The response to treatment of symptoms in breast cancer varies due to its heterogeneity. Breast cancer is divided into several subtypes. In one of the breast cancer types, human epidermal growth factor receptor 2 (HER2) is overexpressed in 20% of cases.^3,4^ The survival time of HER2-positive breast cancer patients is significantly shorter due to the aggressive form of the disease. The amplification of HER2 is reported to have a direct role in the pathogenesis of this cancer. Therefore, the HER2 oncogene is an important target for such cases because it is an important factor in the growth and progression of breast cancer.^4,5^

Radiolabeled trastuzumab (TRZ), a recombinant DNA-derived humanized monoclonal antibody that targets HER2, binds to the HER2 protein, to prevent the epidermal growth factor from reaching the breast cancer cells. Thus, cell division inhibits and the immune system cells easily destroy cancerous cells. TRZ can prolong the progression-free survival time of HER2-positive metastatic breast cancer patients. TRZ is radiolabeled with ^99m^Tc to use in the diagnosis and evaluation of the disease level in patients with HER2-positive breast cancer.^3^

Cancer patients are now treated with chemotherapy, radiotherapy, and/or surgery. Although surgery is the most effective treatment technique, it cannot be applicable in late stages of cancer. In addition, the development of multidrug resistance in cancer patients treated with chemotherapy and serious side effects on healthy tissues in patients receiving radiotherapy led to failure in these treatment methods. For this reason, many studies have been performed to develop more effective and specific drugs or less invasive techniques in cancer diagnosis and treatment.

Nanotechnology research focusing on drug development is currently increasing to obtain a better treatment response for different diseases including cancer.^6−8^ Nanoparticles (NPs) have become popular thanks to their unique size-dependent targeting properties. Recently, many attempts have been made in the development of nanodrug delivery systems in therapeutic, diagnostic, or theranostic areas. Different types of nanosized drug delivery systems such as solid lipid nanoparticles (SLNs), liposomes, polymeric NPs, silica-based NPs, quantum dots, and metal-based nanoparticles containing anticancer drugs have been developed to not only minimize side effects but also increase the efficacy of treatment.^9,10^ Among drug delivery systems, SLNs have numerous advantages, including biocompatibility and a nontoxic profile, scaling-up the production and good carrier systems for hydrophilic and lipophilic drugs. Drug solubility and bioavailability can be enhanced by the encapsulation of drugs in SLNs. In addition, increased treatment response and decreased side effects can be obtained by SLNs thanks to their passively and/or actively targeting and sustained drug release ability. They contain solid lipids and a surfactant that provides the assembly of lipophilic components in aqueous solutions by surrounding the lipid phase.^11,12^ SLNs can be prepared by different production methods such as hot homogenization, cold homogenization, high shear homogenization, and sonication. Herein, high shear homogenization and sonication methods were used to prepare formulations.^13,14^

Nuclear medicine imaging techniques obtain various physiological and functional information regarding the diagnosis and treatment of diseases in the human body.^15,16^ For nuclear medicine, although approximately 50 nanosized systems have been approved by the American Food and Drug Administration (FDA) for therapy, no nanosized system has been approved as an imaging agent to date.^17−20^ Nanoparticles including SLN can be radiolabeled with alpha-, positron-, negatron-, or gamma-emitting radionuclides by different labeling techniques.^16^ Radiolabeled SLNs can be an attractive system as a therapeutic, imaging, or theranostic agent in nuclear medicine. The radiolabeling of SLN with alpha- and negatron-emitting radionuclides provides the obtainment of therapeutic radiopharmaceuticals, whereas positron- and gamma-emitting radionuclides are used to develop imaging agents or theranostic systems. Among radionuclides, ^99m^Tc offers some advantages such as its widespread availability in nuclear medicine clinics, pure and detectable gamma energy by gamma counters, a gamma camera, single-photon emission tomography, and a proper physical half-life to image.^21^

Taking these facts into consideration, TRZ was encapsulated in SLN formulations to obtain sustained drug release and to enhance drug bioavailability via the passive targeting ability of SLN depending on its nanosize. Radiolabeled TRZ-loaded SLN formulations were designed to evaluate their cytotoxicity and apoptosis capacity and to investigate the in vivo pharmacokinetic behaviors. After the preparation of TRZ-loaded SLNs, microscopic imaging, particle size, and zeta potential values were evaluated in terms of characterization studies. Stability studies of formulations were performed under three different storage conditions. Cytotoxicity profiles of formulations were exhibited on MCF-7, MDA-MB-231, and HACAT, and apoptosis effects of formulations were exhibited on MCF-7 cells for breast cancer. Furthermore, they were labeled with ^99m^Tc following the determination of the optimal amount of reducing agent (stannous chloride) in a direct radiolabeling procedure. Additionally, the cellular bindings of SLN formulations in MCF-7, MDA-MB-231, and HACAT cells were evaluated. Last but not least, the pharmacokinetic behavior of TRZ-loaded SLN formulations was investigated in rats. Although nanosized formulations containing TRZ were designed in the literature,^22^ our study is significant in that it is the first study in the literature regarding the in vitro and in vivo evaluation of radiolabeled, TRZ-loaded SLN formulation.

## 2 Materials and Methods

### 2.1 Materials

Lecithin, stearic acid, and 3-[4,5-dimethylthiazol-2-yl]-2,5-diphenyltetrazolium bromide (MTT) were obtained from Sigma-Aldrich (St Louis, MO, USA). TRZ was a gift from Novartis (East Hanover, NJ, USA). The MCF-7 (ATCC) cell line was provided by ATCC (Manassas, VA, USA). Na^99m^TcO4 was acquired from Ege University Nuclear Medicine Department (Izmir, Turkey).

### 2.2 Preparation of Trastuzumab-Loaded Solid Lipid Nanoparticles

Initially, the lipid phase consisting of stearic acid and lecithin mixtures at molar ratios of 8:2 and 6:4, respectively, were heated up to 85 °C into a water bath. One milligram of TRZ was added to a melted lipid mixture. The TRZ-to-lipid phase mass ratios were 1:5 and 1:8 for TRZ-SLN-1 and TRZ-SLN-2 formulations. Simultaneously, 10 mL of distilled water was added to the lipid phase, and the resulting emulsion was homogenized by using a high-speed stirrer at 10 000 rpm for 5 min and further sonicated utilizing a Vibracell tip sonicator at 1000 W and 30 kHz in 20 s changing cycles for 10 min in a cold water bath of 4–5 °C.

### 2.3 Characterization Studies

#### 2.3.1 Scanning Electron Microscope of Formulations

The size and surface properties of TRZ-SLNs were examined under a high vacuum on a scanning electron microscope (Thermo Scientific Apreo S, Waltham, MA, USA). For this purpose, the samples were first coated with 80% gold and 20% palladium at a 7 nm thickness using a Leica EMACE 600 (Leica Microsystems, Wetzlar, Germany) brand coating device. The coating was prepared under a vacuum of 5 × 10^–4^ mbar. The coated samples were scanned at a magnification range of ×50.000 and increased voltage conditions of 5 kV.

#### 2.3.2 Particle Size and Zeta Potential of Formulations

The mean particle size, polydispersity index (PDI), and zeta potential values of the formulations were determined by a Zetasizer (Malvern NanoZS, Malvern Instruments, Malvern, UK).

The mean particle size and PDI values of the formulations were measured with the dynamic light scattering method. The results were obtained by averaging five measurements at an angle of 173° using disposable cells.

The zeta potential of samples was measured using disposable plain folded capillary zeta cells. The Helmholtz–Smoluchowski equation was used to calculate the zeta potential of the formulations from the electrophoretic mobility under an electrical field of 40 V/cm. All measurements were repeated at least five times at 25 ± 2 °C.

#### 2.3.3 Entrapment Efficiency and Loading Capacity of Formulations

The entrapment efficiency (EE) and loading capacity (LC) of drugs to formulations are important properties to achieving accurate administration of a drug delivery system. The EE and LC of TRZ in SLN formulations were performed by using a dialysis bag that is 12–14 kDa in molecular weight. For that, 1 mL of TRZ-SLN formulations was implemented in dialysis bags. The samples were ultracentrifuged at 5000 rev min^–1^ and filtered by using a cellulose nitrate membrane. The amount of TRZ was measured by size exclusion high performance liquid chromatography (SEC-HPLC). The analysis procedure was executed by using SEC-HPLC (PerkinElmer 200; Woodbridge, ON, Canada), a BioSep SEC-s4000 analytical column, and a 100 mM sodium phosphate (pH = 7)/acetonitrile (ACN) mixture (60:40 v/v) as the mobile phase. The flow rate and excitation wavelength are adjusted to 0.5 mL^–1^ and 280 nm.

The EE and LC were calculated according to the following equations:^23,24^
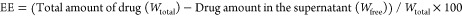

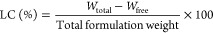


### 2.4 Stability Studies

The stability of SLN formulations at 5 ± 3 °C and 25 ± 5 °C under 60% ± 5% relative humidity (RH) and at 40 ± 5 °C under 75% ± 5% RH of TRZ- loaded formulations were tested for 3 months. The values of particle size, PDI, and zeta potential and the visual appearance of formulations were evaluated. The initial and 3-month values were statistically compared.

### 2.5 Radiolabeling Studies

**Figure 1:**
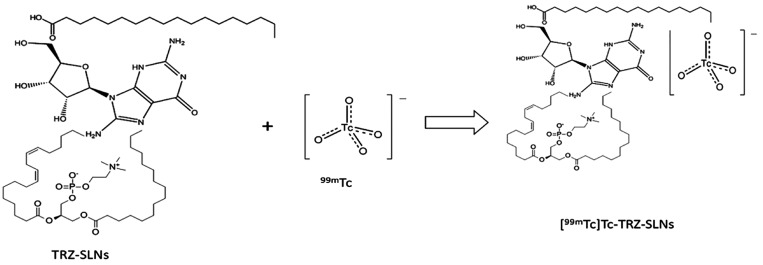
An equation for the reaction of radiolabeling.

The SLN formulations (SLN-1, SLN-2, TRZ-SLN-1, and TRZ-SLN-2) were radiolabeled using 0.1 mL of Na^99m^TcO_4_ solution in saline with 1 mCi radioactivity. The stannous chloride (SnCl_2_) solution in 0.01 N HCl was used as the reducing agent, and four different amounts (10, 100, 500, and 1000 μg) of SnCl_2_ were tested for the detection of the optimum radiolabeling conditions. Briefly, the SnCl_2_ and Na^99m^TcO_4_ solutions were added to 1 mL of SLN formulations (SLN-1, SLN-2) and TRZ-SLN formulations (TRZ-SLN- 1, TRZ-SLN-2) under a bubbling nitrogen atmosphere. Then, the mixtures were vortexed for 60 s and incubated for 30 min at room temperature. The SLN formulations were dialyzed with a dialysis bag with a 3.5 kDa cutoff size for 5 h at 4 °C against phosphate-buffered saline (PBS, pH 7.4) to remove the free ^99m^Tc. An equation for the reaction of radiolabeling was demonstrated in Figure 1.

#### 2.5.1 Quality Control of Radiolabeled Formulations

The radiolabeled formulations were controlled by instant thin-layer chromatography on silica gel-coated fiber sheets (2 × 8 cm^2^) as the stationary phase and acetone and a pyridine/acetic acid/water (PAW, 3:5:1.5) solvent as the mobile phase to separate free pertechnetate and radiocolloids that remained at the origin while the radiolabeled formulation and pertechnetate moved with the solvent front (colloid, Rf = 0.0; radiolabeled formulation, Rf = 1.0). The radioactivity in samples was determined by a well-type of gamma counter (Sesa Uniscaller). The amount of radiolabeled formulation was determined by subtracting the migrated activity with the solvent using the acetone from that of using PAW solvent. The percentage of radiolabeling efficiency (RE %) was calculated using the following equation:
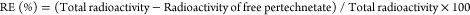


#### 2.5.2 Stability of Radiolabeled Formulations

The stability of radiolabeled formulations was evaluated in saline and a culture medium. For this purpose, 200 μL of radiolabeled formulations were incubated with 800 μL of saline and a cell medium at room temperature for 6 h. The samples were assayed up to 6 h by using a well-type gamma counter (Sesa Uniscaller) to evaluate the in vitro stability of radiolabeling.

### 2.6 Cell Culture Studies

The MCF-7, MDA-MB-321, and HaCat (ATCC, Manassas, VA, USA) cells were grown in Dulbecco’s Modified Eagle’s Medium (DMEM) supplemented with 10% fetal bovine serum and 1% penicillin–streptomycin in a humidified atmosphere with 5% CO_2_ at 37 °C. The cells were cultured in flasks with 25 cm^2^ of surface area until 80%–90% confluence and seeded at a density of 5 × 105 cells per well in six plates.

#### 2.6.1 Cellular Binding of Radiolabeled Formulations

The cellular binding (CB) studies were performed on MCF-7, MDA-MB-231, and HaCat cell lines. The cells were incubated with radiolabeled SLNs (SLN-1, SLN-2, TRZ-SLN-1, and TRZ-SLN-2) and Na^99m^TcO_4_ solution with 1 mCi mL^–1^ radioactivity at 37 °C. After the incubation, the culture medium was removed, and the cells were washed to remove loosely bound surface radioactivity. Radiolabeled SLNs with the same activity were dispersed in the same amount of PBS for control. After the collection of cells by adding 0.5 mL of trypsin–ethylenediaminetetraacetic acid, the radioactivity values of cells and the control were measured by using a gamma counter (Sesa Uniscaller). The CB percentage (CB%) of radiolabeled formulations was calculated using the following formula:
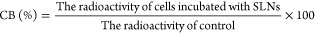


#### 2.6.2 Cytotoxicity Studies

The cytotoxicity of SLNs (SLN-1, SLN-2, TRZ-SLN-1, and TRZ-SLN-2) in MCF-7, MDA-MB-231 (breast cancer), and HaCaT (human keratinocyte) cell lines were evaluated with a MTT cell viability assay. First, the cells were seeded in a 96-well plate at a concentration of 1.2 × 105 cells/mL and incubated with SLN formulations for 24, 48, or 72 h. After an incubation period, 10 L of MTT solution (5 mg mL^–1^) was added to each well for 4 h at 37 °C. Then, 150 L of dimethyl sulfoxide solution was added to obtain dissolution of MTT formazan crystals. The absorbance was read at 570 nm using a microplate reader. Cell viability (%) was calculated using the following formula:
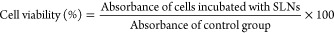


#### 2.6.3 Apoptosis Assay

An apoptosis study was performed by staining cells with acridine orange (AO)/ethidium bromide (EB) solution and Hoechst 33258 dyes. Hoechst 33258 dyes contain specific fluorochromes. The viable and necrotic cells were distinguished under an apoptosis mechanism by using differential staining with specific fluorochromes. In this study, the cell morphology changes were analyzed by Hoechst 33258 dyes. The cells were seeded in 24-well plates after 90% confluence. The developed formulations (SLN-1, SLN-2, TRZ-SLN-1, and TRZ-SLN-2) were incubated with the cells for 48 h at 37 °C. After that, the formulations were removed, and the cells were washed and fixed with pH 7 phosphate buffer for 15 min. Then, 10 μg mL^–1^ Hoechst 33258 solution in pH 7 phosphate buffer was added and maintained at room temperature for 10 min. The morphology and color of cells were evaluated under a fluorescence microscope. The AO/EB dual stain method with several modifications was used to investigate the apoptotic morphology of cells incubated with the SLNs formulation.^25^ Briefly, developed SLNs (SLN-1, SLN-2, TRZ-SLN-1, and TRZ-SLN-2) were administered to cells to show some changes in MCF-7 cells for 24 h. After that, cells were resuspended in pH 7 phosphate buffer and then transferred to glass slides. Twenty microliters of AO/EB solution (3.5 μM AO and 2 μM EB in PBS) was added to the formulations, and they were covered with a coverslip.^26^ Morphological changes were also evaluated and imaged by fluorescence microscopy with a 350–400 nm filter (200× magnification).

### 2.7 In Vivo Studies

All of the experiments and handling of animals were performed in accordance with Helsinki and local law for the protection of animals approval by Universidad Miguel Hernandez. Experiments were performed according to the protocol approved by the Ethics Committee (UMH-DI-MBS-01-14 2014.315.E.OEP Distribution of drug in rat organism and reduction or tumor). To evaluate the in vivo pharmacokinetic performance of 1 mg mL^–1^ of TRZ solution in saline, TRZ-SLN-1, and TRZ-SLN-2, 300–350 g male Sprague–Dawley rats were divided into three groups (*n* = 6). They were provided by Miguel Hernandez University, School of Pharmacy, Animals Research Laboratory, Spain and had free access to food and water. One milligram per milliliter of TRZ-SLN-1 and of TRZ-SLN-2 formulations were administered via the parenteral route. Blood samples (0.3 mL) were collected from the jugular vein at 0, 1, 2, 4, 6, and 24 h and then centrifuged at 10 000 rpm for 10 min. After centrifugation, plasma was separated from blood samples, and TRZ concentration in collected samples was analyzed by HPLC. First of all, 500 μL of methanol was put into the plasma and vortexed for 10 min. The obtained mixture was centrifuged at 4000 rpm for 5 min at 5 °C ± 0.5 °C. The supernatant solution was volatilized under nitrogen gas at 50 °C. A mixture of a phosphate buffer (pH 7) and acetonitrile (60:40, v/v) was used as the mobile phase at a flow rate of 1.0 mL/min. The volatilized samples were dissolved in 100 μL of mobile phase and injected into a BioSep SEC-s4000 column, and the detection was made at 280 nm. The calibration curve was drawn, and linearity was provided in the range of 0.5–30 μg/mL with good linearity (*r*^2^ = 0.998).

#### 2.7.1 Pharmacokinetic Analysis

Pharmacokinetic parameters such as plasma concentration of TRZ after administration (C0) and the area under the curve from time zero to the last measurable concentration, demonstrating the detected exposure to TRZ (AUC0–t), were calculated by using the WinNonlin Program (Version 8.3, Pharsight Co, Mountainview, CA)-noncompartmental analysis.

### 2.8 Statistical Analysis

The statistical analysis was performed by a analysis of variance (ANOVA) program. Differences between results were considered statistically significant when the *p* values were less than 0.05. Results were expressed as mean ± standard deviation (SD).

## 3 Results

### 3.1 Preparation of Trastuzumab-Loaded Solid Lipid Nanoparticles

Recently, various methodologies have been used for the fabrication of SLN, including self-assembly, homogenization, microemulsion, etc.^1,2^ In this study, TRZ-SLN-1 and TRZ-SLN-2 formulations were successfully prepared with homogenization and sonication techniques. Herein, a stearic acid and lecithin mixture was formed with the application of temperature. TRZ was added in a melted lipid mixture. Distilled water was added to the prepared phase, and the resulting emulsion was homogenized and sonicated.

#### 3.1.1 Characterization Properties of Formulations

**Figure 2:**
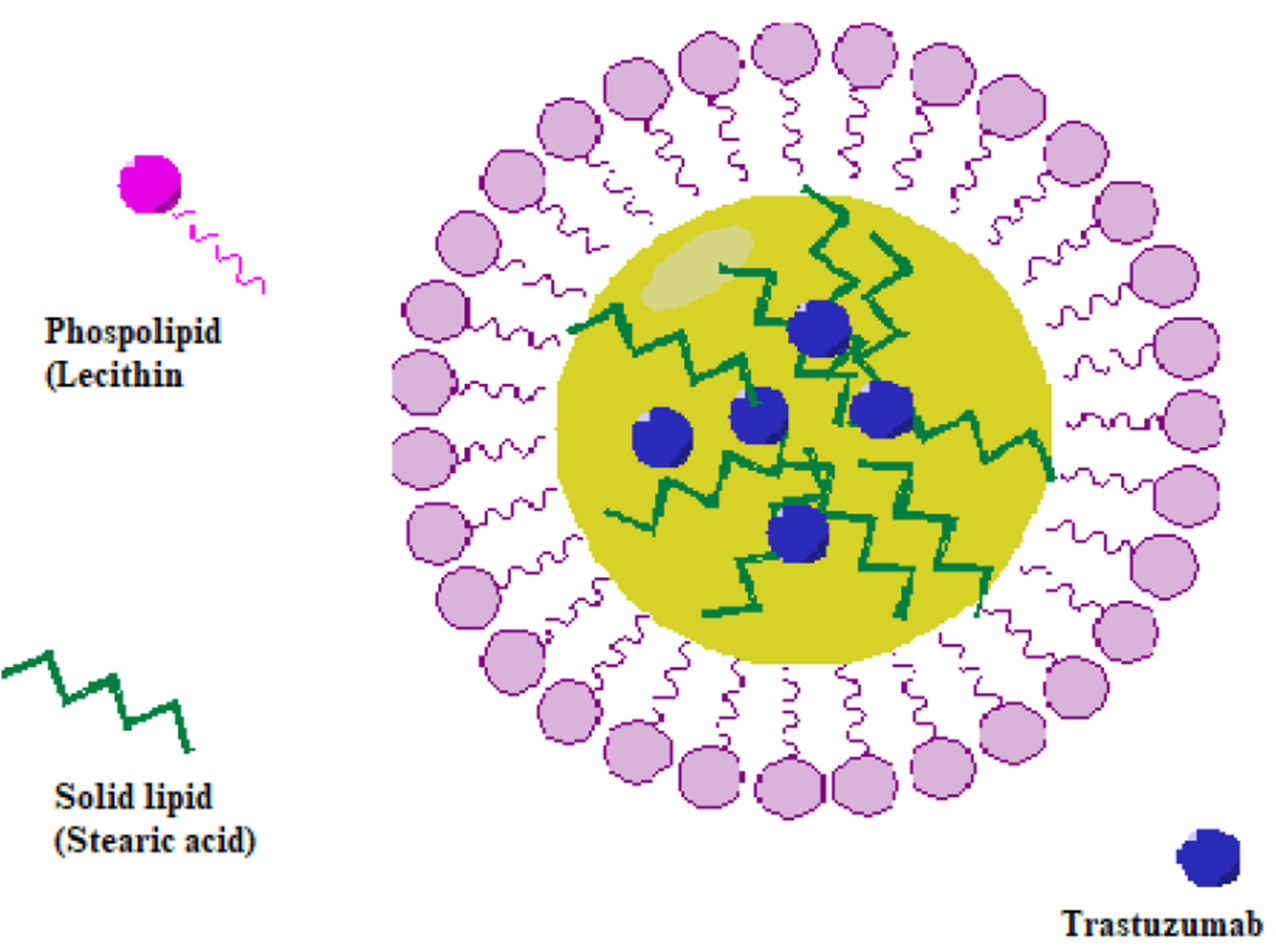
Schematic representation of the preparation of TRZ-loaded SLNs.

**Figure 3:**
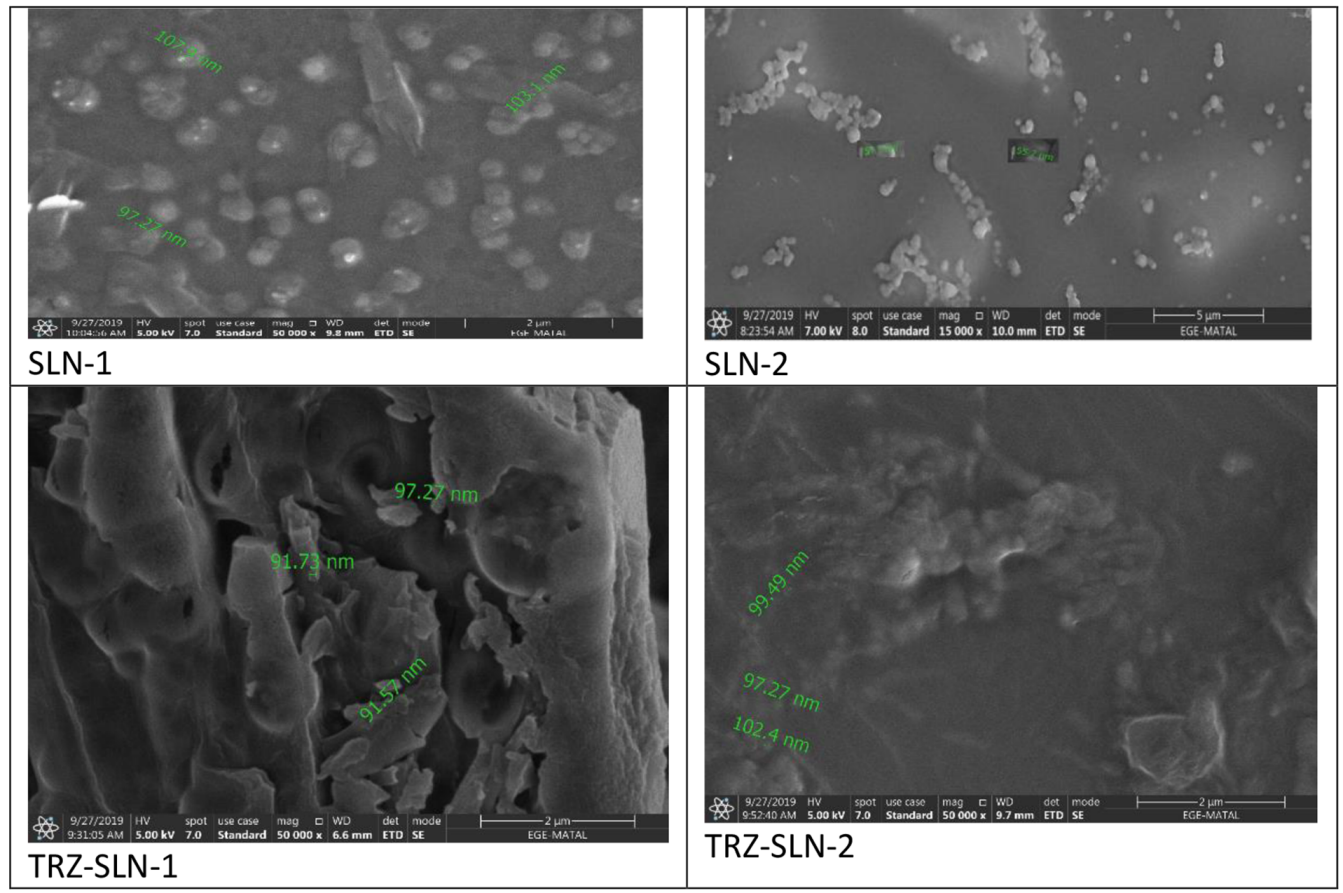
SEM images of SLN formulations.

The TRZ-SLN formulations were successfully prepared by high shear homogenization and sonication techniques. The preparation of TRZ-loaded SLNs with a stearic acid–lecithin mixture as the lipid core is shown schematically in Figure 2. Since the size and the surface charge of SLN would affect the route of the particles inside the living being, it was important to evaluate the characterization of these particles. The particle sizes of all formulations were found below 100 nm, Table 1. The particle sizes of TRZ-SLN-1 and TRZ-SLN-2 formulations were found to be higher than those of empty SLNs (SLN-1 and SLN-2) due to their drug content. The PDI values give information about the particle size distribution of drug delivery systems. The PDI values of SLNs were found between 0.3 and 0.5, as given in Table 1. To determine the surface morphology of the prepared TRZ-SLNs formulation, SEMs were captured. These images are presented in Figure 3. Results from particle size measurement, Table 1, were well supported with image-based microscopic techniques like SEM. Some clusters were encountered in the images which might be connected with the shrinkage of SLNs in concentration in the dispersion medium. It was determined from these images that the majority of the particles had a spherical shape. The developed SLN formulations show the anionic properties due to their zeta potential values between −24 and −28 mV, as given in Table 1. Around −25 mV of zeta potential values in our SLN formulations provide sufficient repelling force and prevent aggregation, thus preserving physical stability. The amounts of TRZ entrapped in the two formulations and loading capacity of these formulations are listed in Table 1. All of them have high EE and LC values, and any statistical difference was observed among formulations (*p* > 0.05). Because lipid carrier systems contain an amorphous structure in the matrix, they are known to transport more drugs than conventional drug delivery systems.

**Table 1:** Characterization Properties of Solid Lipid Nanoparticle Formulations (*p* < 0.05)

formulations	mean particle size (nm) ± SD*	PDI* ± SD*	zeta potential (mV*) ± SD*	entrapment efficiency (% ± SD)	loading capacity (% ± SD)
SLN-1	84 ± 3.1	0.5 ± 0.1	–24 ± 3.8		
TRZ-SLN-1	95 ± 2.3	0.4 ± 0.1	–28 ± 2.2	95.17 ± 3.36	95.96 ± 1.8
SLN-2	83 ± 5.1	0.4 ± 0.1	–23 ± 1.7		
TRZ-SLN-2	97 ± 2.1	0.3 ± 0.2	–25 ± 1.5	93.29 ± 2.48	95.19 ± 0.85

### 3.2 Stability of SLNs

The stability of our formulations in three different storage conditions were checked by the evaluation of the particle size, PDI, and zeta potential of formulations for 3 months. To mimic the refrigerator and room condition storage, formulations were kept at +5 °C and 25 ± 2 °C under 60% ± 5% RH, respectively. Moreover, the temperature of 40 ± 2 °C and 75% ± 5% RH were chosen for the accelerated conditions.^27^ Physicochemical characterization values of SLNs at the first day and after 3 months of storage are given in Table 2. Any visible changes of clarity and aggregation were detected in the formulations after 3 months of storage under different conditions. In addition, at the end of the 3 months, the alterations in particle size, PDI, and zeta potential values of SLNs were found to be statistically insignificant, compared with the initial values. In short, all SLN formulations exhibited stable profiles at three different conditions for 3 months.

**Table 2:** Stability Results of SLN Formulations

	initial values			3 months values								
	25 °C			5 °C			25 °C			40 °C		
formulations	particle size nm ± SD*	PDI ± SD*	zeta potential (mV*) ± SD*	particle size nm ± SD*	PDI* ± SD*	zeta potential (mV*) ± SD*	particle size nm ± SD*	PDI* ± SD*	zeta potential (mV*) ± SD*	particle size nm ± SD*	PDI* ± SD*	zeta potential (mV*) ± SD*
SLN-1	84 ± 3.1	0.5 ± 0.1	–24 ± 3.8	89 ± 2.3	0.4 ± 0.1	–25 ± 2.9	85 ± 5.7	0.4 ± 0.1	–25 ± 2.6	88 ± 3.8	0.3 ± 0.1	–26 ± 2.8
TRZ-SLN-1	95 ± 2.3	0.4 ± 0.11	–28 ± 2.2	97 ± 1.1	0.5 ± 0.1	–29 ± 3.1	90.23 ± 4.5	0.3 ± 0.1	–29 ± 1.1	97 ± 1.1	0.4 ± 0.1	–27 ± 3.9
SLN-2	83 ± 5.1	0.4 ± 0.1	–23 ± 1.7	90 ± 1.8	0.3 ± 0.1	–24 ± 5.4	87 ± 2.1	0.4 ± 0.1	–24 ± 1.3	87 ± 2.4	0.5 ± 0.1	–25 ± 0.9
TRZ-SLN-2	117 ± 2.1	0.3 ± 0.2	–25 ± 1.5	99 ± 1.1	0.4 ± 0.1	–26 ± 1.3	126 ± 3.3	0.3 ± 0.2	–26 ± 2.7	119 ± 0.9	0.4 ± 0.1	–27 ± 1.3

### 3.3 Results of Radiolabeling

**Figure 4:**
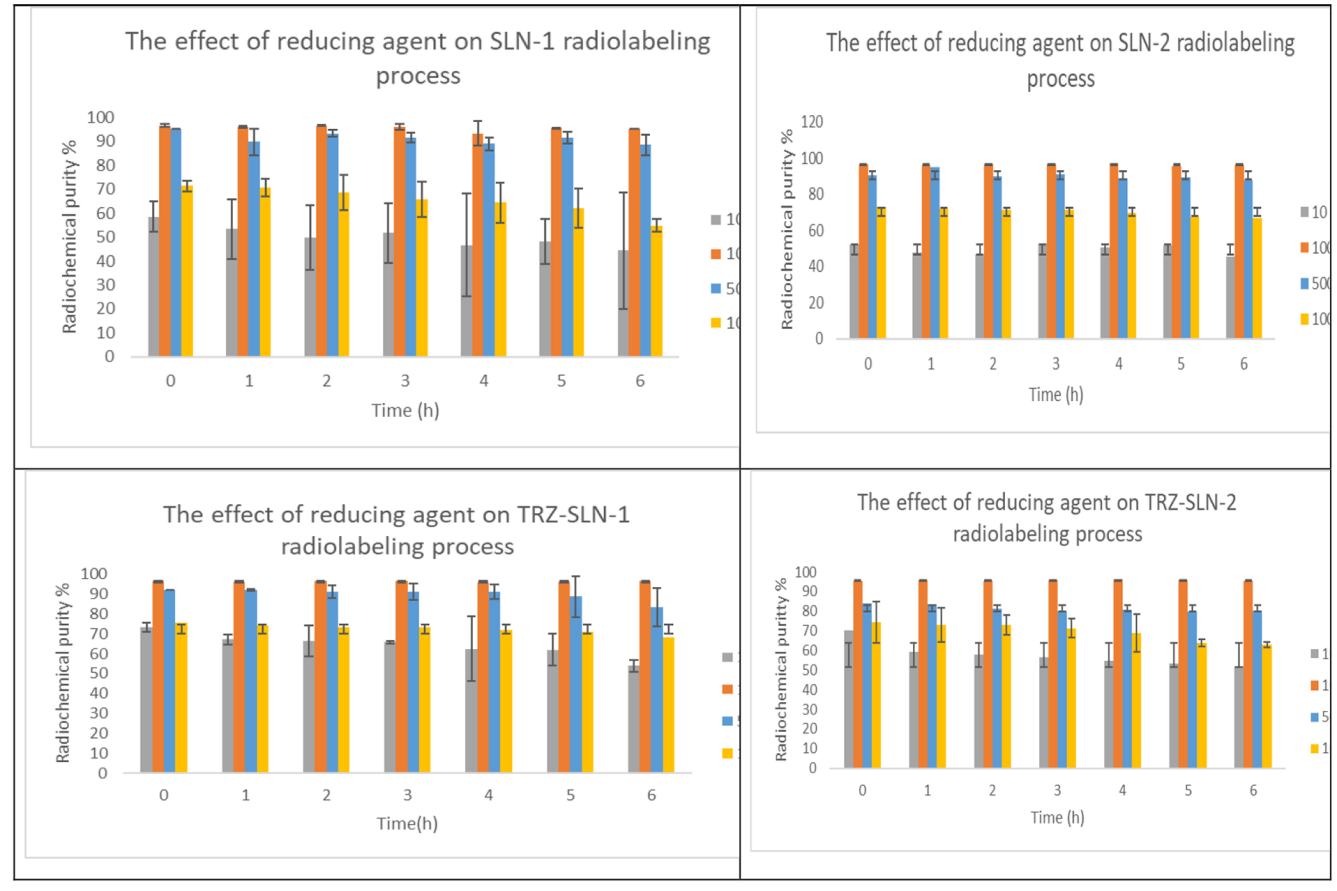
Effect of the amount of SnCl_2_ on the radiolabeling process of SLNs (*n* = 3).

The effect of different amounts of SnCl_2_ on the radiolabeling of SLNs was evaluated to determine the optimal concentration. As given in Figure 4, the highest radiolabeling efficiencies for all formulations were obtained by using 100 μg of SnCl_2_ compared with other amounts (*p* < 0.05). Our finding is like previous studies that detected different amounts of reducing agents changing from 50 to 1000 as the optimal concentration in the literature.^28,23^ Moreover, the radiochemical purities of all SLN formulations were found to be higher than 90% at different time intervals for 6 h. This result presents that radiolabeling of SLNs was stable during at least the physical half-life of ^99m^Tc.

### 3.4 Radiolabeling Stability

**Figure 5:**
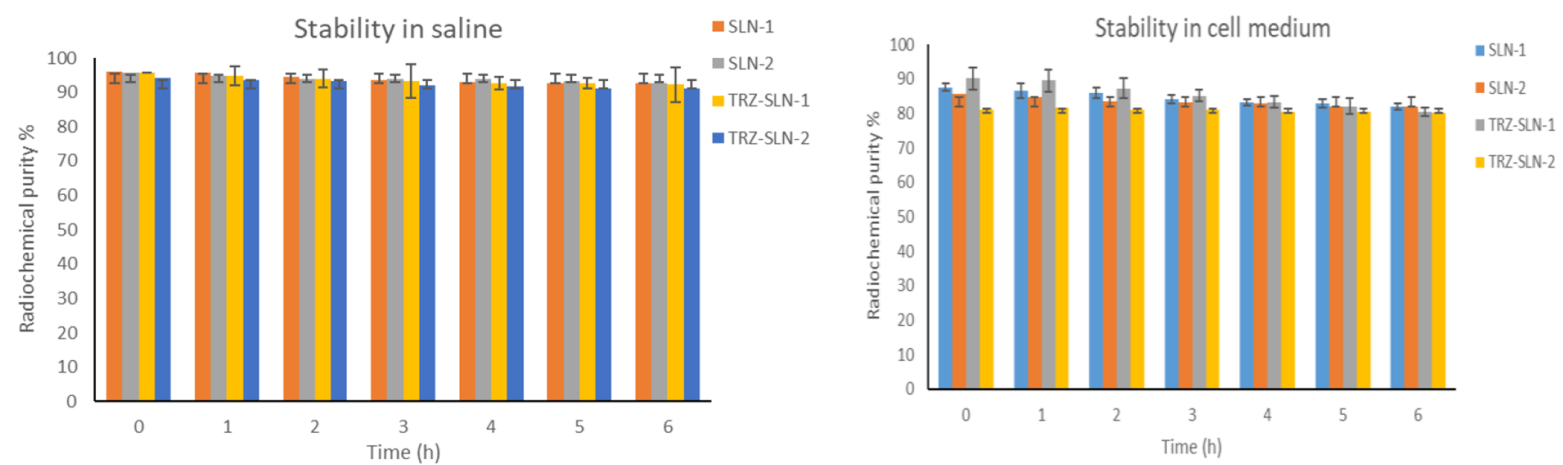
Stability of in vitro radiolabeling of SLNs in saline and cell medium (*n* = 3).

Furthermore, the binding of radionuclide and pharmaceutical parts in radiopharmaceuticals should present in vivo stability to reach the target area in the body. Therefore, ^99m^Tc labeled-SLNs complexes were also evaluated in terms of radiolabeling stability by using different media. The stabilities of radiolabeled SLN formulations in saline and cell culture medium are given in Figure 5. The radiochemical purity of radiolabeled SLN formulations was between 80.01% ± 1.09% and 90.13% ± 3.12% in the cell culture medium and 91.16% ± 5.15% and 96.1% ± 3.73% in saline.

### 3.5 Cellular Binding of Radiolabeled SLNs

The CBs (%) of radiolabeled formulations and Na^99m^TcO4 were calculated by measuring the radioactivity in MCF-7, MDA-MB-231, and HaCat cell lines. As given in Table 3, higher CB values were obtained in the cells incubated with SLNs compared to those with Na^99m^TcO_4_, and the highest CB (%) was obtained from cells incubated with TRZ-SLN-1 for 2 h in the MCF-7 cell line. This finding suggested that the SLN formulations increased the cellular uptake compared with the CB (%) of free technetium. In addition, the CB (%) of radiolabeled TRZ-SLN-1 and TRZ-SLN-2 formulations was found to be significantly higher compared with SLN-1 and SLN-2 formulations thanks to the targeting ability of TRZ on MCF-7 and MDA-MB-231 cell lines (*p* < 0.05). However, there was no significant difference between formulations in terms of CB (%) in the HaCat cell line (*p* > 0.05).

**Table 3:** Cell Binding Ratio of Radiolabeled Formulations

cell line	time (min)	Na^99m^TcO_4_	SLN-1	SLN-2	TRZ-SLN-1	TRZ-SLN-2
MCF-7	60	57.5 ± 8.6	60.9 ± 2.6	60.9 ± 8.8	67.0 ± 5.8	63.7 ± 6.5
MCF-7	120	59.2 ± 2.9	61.6 ± 1.2	63.7 ± 3.7	72.6 ± 0.9	69.3 ± 3.3
MDA-MB-231	60	45.2 ± 3.2	48.6 ± 3.5	53.5 ± 2.5	58.6 ± 2.1	62.9 ± 4.1
MDA-MB-231	120	49.5 ± 2.5	57.3 ± 1.9	56.7 ± 1.8	68.4 ± 4.3	70.8 ± 2.1
HaCat	60	10.2 ± 0.7	33.4 ± 1.4	33.1 ± 3.9	30.9 ± 3.6	34.2 ± 2.6
HaCat	120	12.1 ± 2.5	32.7 ± 2.2	40.5 ± 4.3	35.3 ± 1.8	38.7 ± 3.3

### 3.6 Cytotoxicity of SLNs

**Figure 6:**
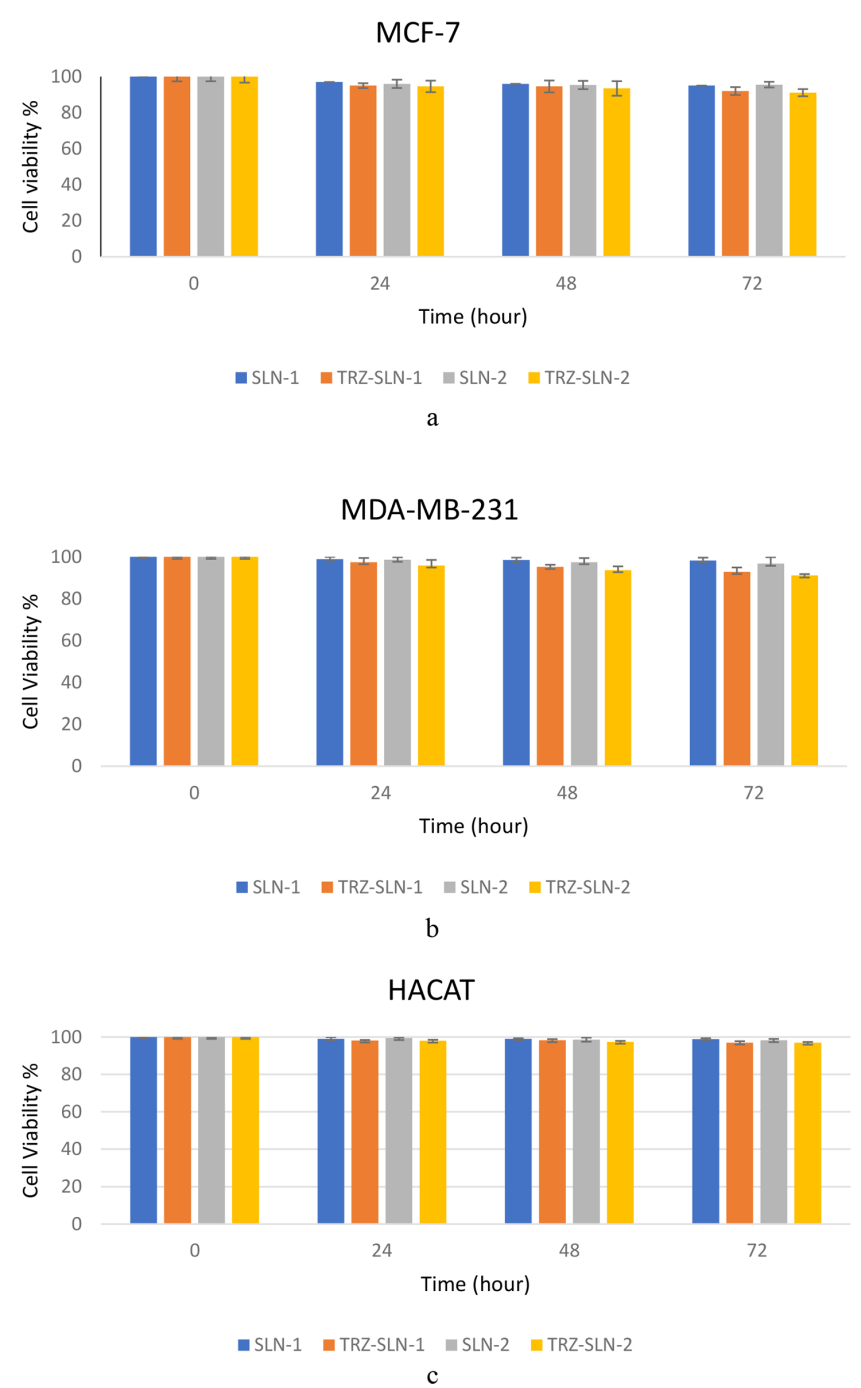
Cell viability of SLNs on MCF-7 cells (a), MDA-MB-231 (b), and HACAT (c).

The cell viabilities were higher than 90% for all formulations at 24, 48, and 72 h time points Figure 6. Although TRZ-SLN-1 and TRZ-SLN-2 formulations exhibited a slightly higher cytotoxic effect than SLN-1 and SLN- 2 due to their TRZ content, this difference is statistically insignificant (*p* ≥ 0.05). These findings suggest that SLNs did not cause toxic effects on healthy cells due to the biocompatible profile of SLNs.

### 3.7 Apoptotic Activity of SLNs

**Figure 7:**
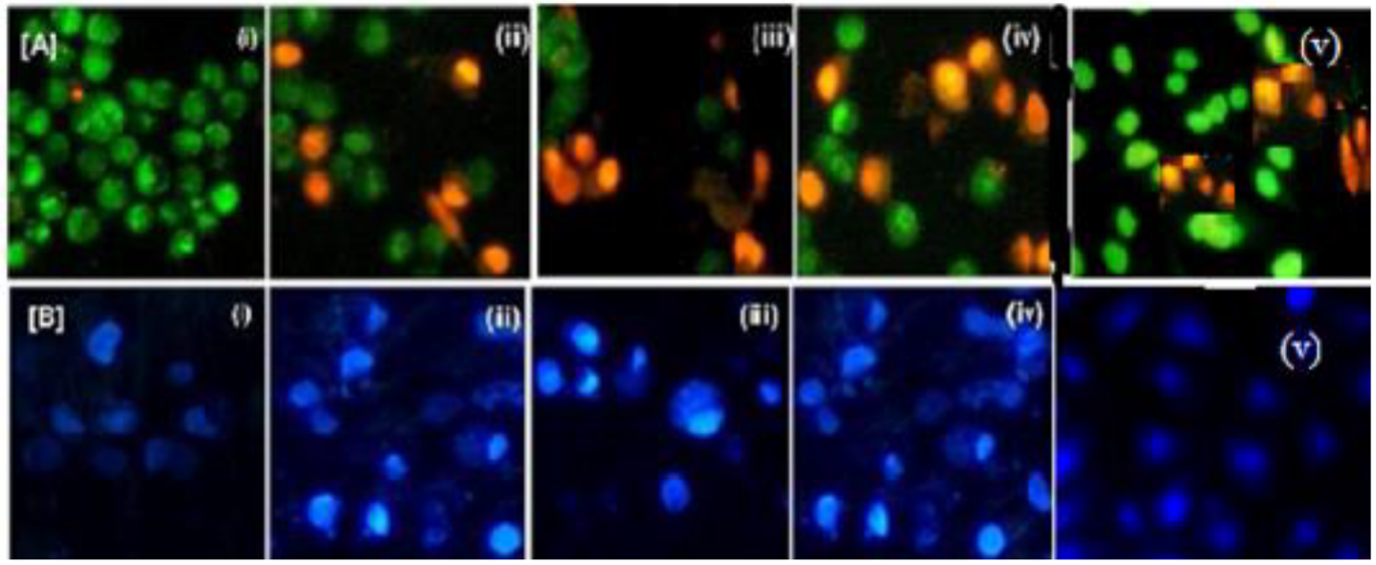
Morphological changes during apoptosis of MCF-7 cells on (A) AO/EB and (B) Hoechst staining images. (i) Control, (ii) SLN-1, (iii) SLN-2, (iv) TRZ-SLN-1, and (v) TRZ-SLN-2.

The apoptotic activity induction of SLN formulations (SLN-1, SLN-2, TRZ-SLN-1, and TRZ-SLN-2) on MCF-7 cells was shown in terms of cell viability or control cells, Figure 7. While the cells are classified viable cells and they experienced unbroken chromatin with a green fluorescing nucleus in Figure 7A,i, the early apoptotic cells were observed in Figure 7A,ii–v because fragmentation of DNA had started, the undamaged cell occurred, and the formation of chromatin with green fluorescing nuclei had been obtained. While DNA and chromatins were damaged with orange to red fluorescing nuclei, the necrotic cells were also observed with a swollen cell membrane structure. Also, DNA and chromatin fragmentations were not seen in necrotic cells. On the basis of Figure 7A, all SLN formulations induced the apoptosis activity compared to the control group, and the highest apoptotic activity induction was observed on cells incubated with TRZ-SLN-1 and TRZ-SLN-2 thanks to their TRZ content. The cytological changes in the MCF-7 cells were evaluated after administration of SLN formulations for 24 h. The alterations of normal and abnormal cells were separated by using Hoechst 33258 staining that is a specific reference for the nucleus core and cytoplasm at the primary level to define apoptosis (Figure 7B).^29^ In this study, changes in the cell cytology were observed because of variations in the structure of the nucleus core and cytoplasm (Figure 7B,ii–iv). According to these results, TRZ-SLN-1 and TRZ-SLN-2 formulations exhibited some changes on MCF-7 cells, and the formulations can potentially be for further in vivo studies for the treatment of breast cancer.

### 3.8 In Vivo Studies

**Figure 8:**
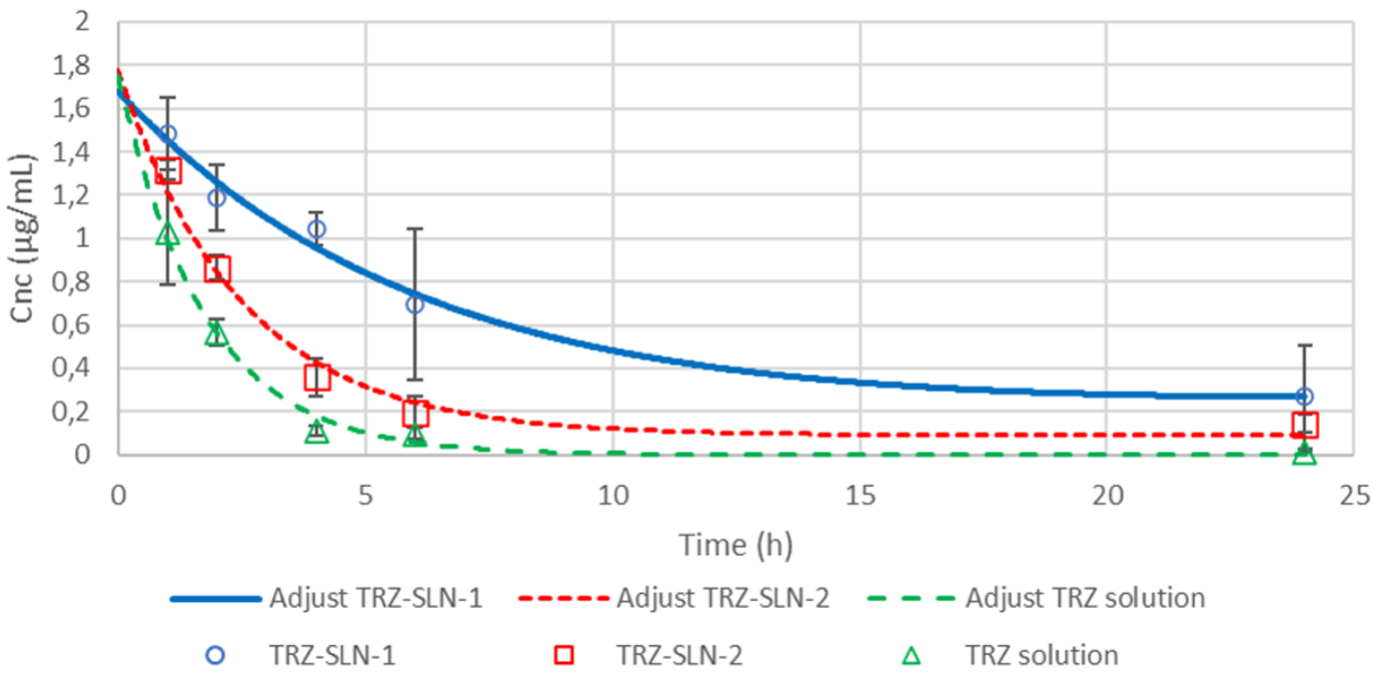
Plasma concentration–time profiles for the prepared SLN formulations.

TRZ concentration in collected samples was analyzed by SEC-HPLC. The recovery of TRZ after spiking of blank plasma is found to be 93%. The plasma profiles of TRZ solution, TRZ-SLN-1, and TRZ-SLN-2 are shown in Figure 8. The major pharmacokinetic parameters, which are C0 and AUC0–t, were calculated for TRZ solution, TRZ-SLN-1, and TRZ-SLN-2 and represented are in Table 4. C0 was found to be 1.102 μg mL^–1^, 1.32 μg mL^–1^, and 1.48 μg mL^–1^ for TRZ solution, TRZ-SLN-2, and TRZ-SLN-1 formulations. The highest C0 value was obtained with TRZ-SLN-1, and it is confirmed that the TRZ-SLN-1 formulation showed a significantly higher plasma concentration compared to TRZ-SLN-2 formulation and TRZ solution (*p* < 0.05). Any initial burst release was not observed, followed by a slow-release period in all formulations. This release profile can be described by the drug enriched shell model.^29^ As given in Table 4, the AUC0–24, i.e., the amount of drug absorbed in the blood circulation, was also significantly (*p* < 0.05) higher for the TRZ-SLN-1 formulation (13.94 ± 6.03 μg/mL h) when compared to TRZ-SLN-2 (6.01 ± 1.15 μg/mL h) and TRZ solution (3.12 ± 1.19 μg/mL h). TRZ-SLN-1 has a 1:5 TRZ-to-lipid phase mass ratio, and the lipid ratio of formulations has affected the release of TRZ. Furthermore, the negative surface charge (28 ± 2.2 mV) and smaller particle size (95 ± 2.3 nm) of the TRZ-SLN-1 seems to greatly enhance the TRZ concentration. According to these results, the improvement of the pharmacokinetic profile of TRZ was carried out with TRZ-SLN-1 formulation in comparison with, especially, TRZ solution.

**Table 4:** Pharmacokinetic Parameters of Prepared SLN Formulations

pharmacokinetic parameters	TRZ solution	TRZ-SLN-1	TRZ-SLN-2
C0 (ug/mL)	1.102 ± 0.17	1.48 ± 0.18	1.32 ± 0.04
AUC0–24 (μg/mL h)	3.12 ± 1.19	13.94 ± 6.03	6.01 ± 1.15

## 4 Discussion

Encapsulation of peptides in drug delivery vehicles is a common strategy used in parenteral drug delivery to overcome low solubility, proteolysis, and poor intestinal permeability and to improve storage stability. TRZ is a monoclonal antibody used to treat and target breast cancer. On the basis of these considerations, SLN formulations containing TRZ were developed, and the cell binding capacity, apoptosis mechanism, and cytotoxicity of radiolabeled TRZ-SLNs for breast cancer cells via in vitro and in vivo studies were investigated.

Developed formulations were characterized in terms of particle size, zeta potential, PDI, EE, and LC values. Particle sizes of drug delivery systems play a critical role for their nonspecific accumulation in the tumor tissue by the enhanced permeability and retention (EPR) effect. For EPR phenomena, drug delivery systems should escape from the reticuloendothelial cells and remain for a longer time in blood circulation. Nanoparticles averaging ∼100 nm have been reported to be the most suitable for extending the blood circulation half-life^30^ Therefore, developed SLN formulations were found to be proper in terms of particle size (Table 1) for remaining longer in circulation and nonspecifically targeting the tumor via the EPR effect. According to the literature, the PDI value should be under 0.2 to obtain a monodispersed formulation.^31^ Therefore, SLN formulations have midrange polydispersity (Table 1).

Zeta potential, expressing the electrokinetic potential of colloidal systems, is another key parameter in the stability of SLNs and affects the recognition of the particles via the reticuloendothelial system. It can be defined as the potential difference between a solid surface and its liquid medium. The composition of SLN formulation directly affects the zeta potential of particles.^32^ Our SLN formulations show the anionic properties due to their zeta potential values between −24 and −28 mV, as given in Table 1. Notably, most of the commercially available lipid drug formulations approved by the FDA are negatively charged lipid particulate systems.^33,34^ Moreover, it was reported in the literature that the nanodispersions having around −30 or +30 mV show good physical stability due to a sufficient repulsive force of nanoparticles. Particle aggregation and flocculation were observed in nanodispersions with a small zeta potential value.^35^ Around a −25 mV zeta potential value in our SLN formulations provides a sufficient repelling force and prevents aggregation, thus preserving the physical stability.

Obtaining desired values for EE and LC prevents the dose-dependent side effects, thus improving patient compliance.^36^ SLN was preferred as a lipid-based drug delivery system to provide more TRZ drug encapsulation.^37^ The obtained results demonstrate that the composition of developed formulations is suitable for the delivery of TRZ.

All of the pharmaceutical products should be stable during their shelf lives. Storage conditions have a critical role in the stability of colloidal systems including SLNs. The optimal storage conditions should be specified for them to avoid degradation and coalescence.^38^ As shown in Table 2, particle sizes, zeta potentials, and PDI values of the formulations are different from each other (*p* < 0.05), but these values of each formulation remained the same (*p* > 0.05) during storage conditions and time intervals. According to these results, all SLN formulations were found to be stable at even high temperatures and humidities.

In radiolabeling studies, all SLN formulations were successfully radiolabeled by a tin-reduction direct labeling method, like the study.^39^ The radiochemical purity of radiopharmaceuticals should be over 80% as the basic principle. In the radiolabeling with ^99m^Tc, the oxidation state of ^99m^Tc should be reduced to a +5 oxidation state by using a reducing agent such as ascorbic acid, ferrous ions, or stannous chloride (SnCl_2_). While doing so, the major radiochemical impurities, including radiocolloids, occur by the adsorption of Sn over ^99m^TcO_2_, and free ^99m^TcO_4_^–^ can occur. Therefore, the amount of reducing agent plays an important role not only in obtaining sufficient reduction but also in avoiding the radiocolloid form of ^99m^Tc. The radiolabeling efficiency of all SLN formulations was found higher than 90% at different time intervals for 6 h. This result illustrates that radiolabeling of SLNs was stable during at least the physical half-life. The radiolabeling results are in agreement with those of previous studies performed on radiolabeled SLNs (Figure 4).^40^ Although higher radiolabeling stability values were obtained in saline than in the cell culture medium (Figure 5), this difference showed no statistical significance (*p* ≥ 0.05). In this study, all of the radiolabeled SLN formulations were stable in saline and cell culture media with radiolabeling efficiencies of over 80%, and these results showed that radiolabeled SLN formulations are proper for CB studies.

A high targeting cell binding ratio of radiolabeled systems is critical in clinical administrations. It can affect the quality of target organ images and be localized in the nontargeting organs and cause injury in these tissues. According to in vitro cell binding studies, radiolabeled TRZ-SLN-1 and TRZ-SLN-2 showed a higher cell binding ratio on MCF-7 and MDA-MB-231 cell lines when compared to other formulations (Table 3). Also, the cell binding ratio has been changed at time intervals. Despite the exact reason not being explained, these changes may be due to the pharmaceutical and radionuclide parts of radiolabeled system, affinity of radiolabeled formulations to cells, and time. In the HaCaT cell line, however, radiolabeled TRZ-SLN-1 and TRZ-SLN-2 did not have a higher cell binding ratio compared to other formulations (Table 3)

Apoptotic activity plays a critical role in some normal and pathologic conditions beginning from embryologic development and ending at death. Apoptosis is initiated by morphological changes at the cell membrane, surface organelles, and nucleus. Herein, we tried to indicate the effect of developed formulation administration on MCF-7 cells for 24 h. The results exhibited that some changes of cell organelles in breast cancer are thanks to the delivery of TRZ with SLNs (Figure 7). The SLNs can be uptaken by cells using both endocytic and nonendocytic different mechanisms, such as receptor-mediated endocytosis, diffusion inside the cytoplasm, and fusion or adsorption to the cellular membrane due to the type of SLN.^41,42^ Actively receptor-targeted SLN generally can be internalized by receptor-mediated endocytosis, but untargeted SLNs, including our formulation, are uptaken by fusion or adsorption owing to their similar structure to cellular membranes. The morphological variations form in the cytoplasm and Golgi of cells with chromatin. These changes were assessed during the apoptosis mechanism, and the cells were categorized as follows: Viable cells, early apoptotic cells, late apoptotic cells, and necrotic cells.^43^

The in vitro cytotoxic profiles of SLN-1, SLN-2, TRZ-SLN-1, and TRZ-SLN-2 formulations on the MCF-7, MDA-MB-231, and HaCat cells are tested by the evaluation of cell viability at the end of the MTT test (Figure 6). The similar structures of formulations to cell membranes due to phospholipids and solid lipid contents provide biocompatible, biodegradable, nontoxic, and nonpyrogenic profiles.^44^ In our studies, lipid-based formulations containing cancer drugs showed no cytotoxic effects on human epithelial carcinoma cell lines; these findings are in agreement with our results.^40,45^ Therefore, TRZ-loaded SLN formulations can be considered safe and valuable drug delivery systems for healthy cells in the diagnosis and treatment of breast cancer for future in vivo studies due to their high biocompatibility and nontoxic profiles. When used in the diagnosis of breast cancer, the prepared TRZ-SLN formulations will be radiolabeled with ^99m^Tc, and their toxic effect on cells will be reduced. Given that no pharmacological and toxic effects are desired in radiopharmaceuticals used in diagnosis, the TRZ-SLN formulations prepared will be advantageous for diagnostic and therapeutic applications.

Generally, the undamaged cells with uniform chromatin appear in green color, and this proves that the cells have no apoptotic changes. Liu et al.^46^ explained the importance of the dual AO/EB staining method to define DNA damage and distinguish normal, early apoptotic, late apoptotic, necrotic cells’ structure and morphology. Herein, TRZ-SLN-1 and TRZ-SLN-2 formulations have desired apoptosis induction activity, which also shows potential for further in vivo studies for the treatment of breast cancer (Figure 7).

Among developed formulations, the better pharmacokinetic parameters are provided in TRZ-SLN-1 formulation (Figure 8 and Table 4). This is probably related to the slower elimination and degradation of SLN as a virtue of the small particle size of the lipid nanocarriers and their escape from filtration at the venous sinuses of the spleen. Thus, SLNs within the size range below 100 nm, exhibit the desired residence time in blood circulation.^47^ The lipid excipients existing in the SLN structure protect the encapsulated drug against chemical and enzymatic degradation and alter the release amount of drug.^48^

Tao et al.^49^ and Elbahwy et al.^50^ developed enrofloxacin-loaded docosanoic acid SLNs and glibenclamide-SLNs, respectively. The formulations showed different plasma concentration time curves, sustained release profiles, and better pharmacokinetic parameters in blood circulation when compared to solution form. It could be concluded that sustained drug release from the lipid matrix occurred through dissolution and diffusion and physicochemical properties of the formulations such as particle size. The sustained release profiles for TRZ were observed in TRZ-SLN-1 and TRZ-SLN-2 formulations, too. The sustained release forms release enough of the drug to produce a therapeutic effect. The drug continues to be released in the body at a rate sometimes unequal to the rate of elimination. Especially, lipophilic matrix formulations cause sustained release patterns for drugs. Herein, TRZ-SLN-1 and TRZ-SLN-2 formulations are planned to be used as theranostic formulations and their sustained release capability will strengthen the theranostic feature.

## 5 Conclusions

In this study, TRZ-loaded, ^99m^Tc labeled SLN formulations were developed as a potential theranostic agent for breast cancer. The SLN formulations exhibited proper characterization properties with their nanosize, zeta potential, and PDI values. According to the results of the stability study, all SLN formulations exhibited excellent stability up to 90 days of storage even at 40 °C. In addition, after the determination of the optimal amount of reducing agent as 100 μg of SnCl_2_ for radiolabeling studies, SLN formulations were radiolabeled by a simple and direct radiolabeling technique with a radiochemical purity over 90%. All ^99m^Tc labeled SLN formulations exhibited high radiolabeling stability in different media. Thanks to the characterization properties and stable radiolabeling of ^99m^Tc -SLN formulations, high cellular uptake values on breast cancer cells were obtained. Besides obtaining a biocompatible profile with all SLN formulations, TRZ-SLN-1 and TRZ-SLN-2 formulations exhibited higher apoptosis induction activity on breast cancer cells. The pharmacokinetics of TRZ-loaded SLNs were evaluated in the rat model using a noncompartmental method after a single dose parenteral administration. The TRZ-SLN-1 showed an increase in C0 and AUC0–24 compared to TRZ-SLN-2 and TRZ solution.

In light of the data obtained within this study, TRZ-loaded, ^99m^Tc radiolabeled theranostic SLN formulations were evaluated as promising nanotheranostic agents depending on their characterization profiles, in vitro cellular uptake, biocompatible profiles, apoptosis induction, and pharmacokinetic activities.
